# The role of MSCs and CAR-MSCs in cellular immunotherapy

**DOI:** 10.1186/s12964-023-01191-4

**Published:** 2023-08-01

**Authors:** Lun Yan, Jing Li, Cheng Zhang

**Affiliations:** Medical Center of Hematology, State Key Laboratory of Trauma, Burn and Combined Injury, Xinqiao Hospital, Army Medical University, Chongqing, 400037 China

**Keywords:** Mesenchymal stem cell, CAR, CAR-T cells, CAR-NK cells, CAR-Ms, CAR-DCs, Exosome, Immunomodulation, Immunotherapy

## Abstract

Chimeric antigen receptors (CARs) are widely used by T cells (CAR-T cells), natural killer cells dendritic cells and macrophages, and they are of great importance in cellular immunotherapy. However, the use of CAR-related products faces several challenges, including the poor persistence of cells carrying CARs, cell dysfunction or exhaustion, relapse of disease, immune effector cell-associated neurotoxicity syndrome, cytokine release syndrome, low efficacy against solid tumors and immunosuppression by the tumor microenvironment. Another important cell therapy regimen involves mesenchymal stem cells (MSCs). Recent studies have shown that MSCs can improve the anticancer functions of CAR-related products. CAR-MSCs can overcome the flaws of cellular immunotherapy. Thus, MSCs can be used as a biological vehicle for CARs. In this review, we first discuss the characteristics and immunomodulatory functions of MSCs. Then, the role of MSCs as a source of exosomes, including the characteristics of MSC-derived exosomes and their immunomodulatory functions, is discussed. The role of MSCs in CAR-related products, CAR-related product-derived exosomes and the effect of MSCs on CAR-related products are reviewed. Finally, the use of MSCs as CAR vehicles is discussed.

**Graphical Abstract:**

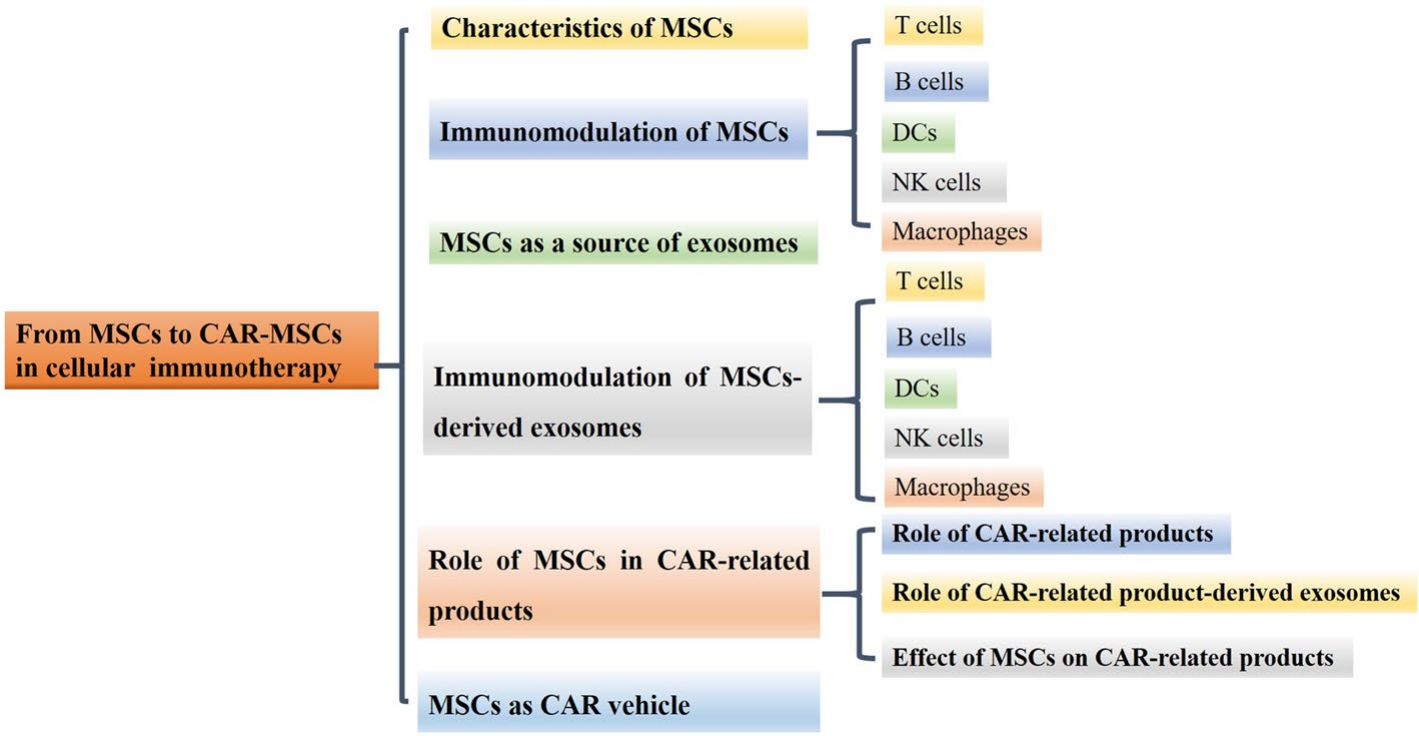

Video Abstract

**Supplementary Information:**

The online version contains supplementary material available at 10.1186/s12964-023-01191-4.

## Introduction

Cellular immunotherapy, as a novel method, has a dynamic role in targeted therapies [1–6]. The use of the chimeric antigen receptor (CAR) has been widely implemented in T cells (CAR-T cells), natural killer cells (CAR-NK cells), dendritic cells (CAR-DCs) and macrophages (CAR-Ms) [1]. CARs can independently recognize tumor-associated antigens (TAAs) in the major histocompatibility complex (MHC) [2–6]. An unprecedented response rate was achieved following the use of CAR-T cells in the treatment of refractory B-cell malignancies [2–5]. Nevertheless, there are several challenges in the use of CAR-related products, including relapse of the disease, the poor persistence of cells carrying CARs, cell dysfunction or exhaustion, low efficacy against solid tumors, immunosuppression by the tumor microenvironment, immune effector cell-associated neurotoxicity syndrome (ICANS) and cytokine release syndrome (CRS) [7–10]. Therefore, new strategies should be explored.

Mesenchymal stem cells (MSCs), which exhibit multilineage differentiation and self-renewal functions, can be isolated from a variety of sources, such as adipose tissue, umbilical cord tissue, amniotic fluid, and bone marrow [11, 12]. Recent studies have shown that MSCs can improve CAR-T-cell activity and deliver oncolytic immunotherapy to improve the antitumor activity of CAR-T cells [13, 14]. It is likely that MSC-derived exosomes can play the same role as MSCs [15]. MSCs can also secrete a variety of cytokines and chemokines, which makes them an attractive complement to cellular immunotherapy [16]. Therefore, the function of MSCs indicate that they have the ability to be a biological vehicle for CARs. In this review, we first discuss the characteristics of MSCs and their immunomodulatory functions. Then, the role of MSCs as a source of exosomes, including the characteristics of MSC-derived exosomes and their immunomodulatory functions, is discussed. The role of MSCs in CAR-related products and CAR-related product-derived exosomes and the effect of MSCs on CAR-related products are reviewed. Finally, the use of MSCs as CAR vehicles is discussed.

### Characteristics of MSCs

**Fig. 1:**
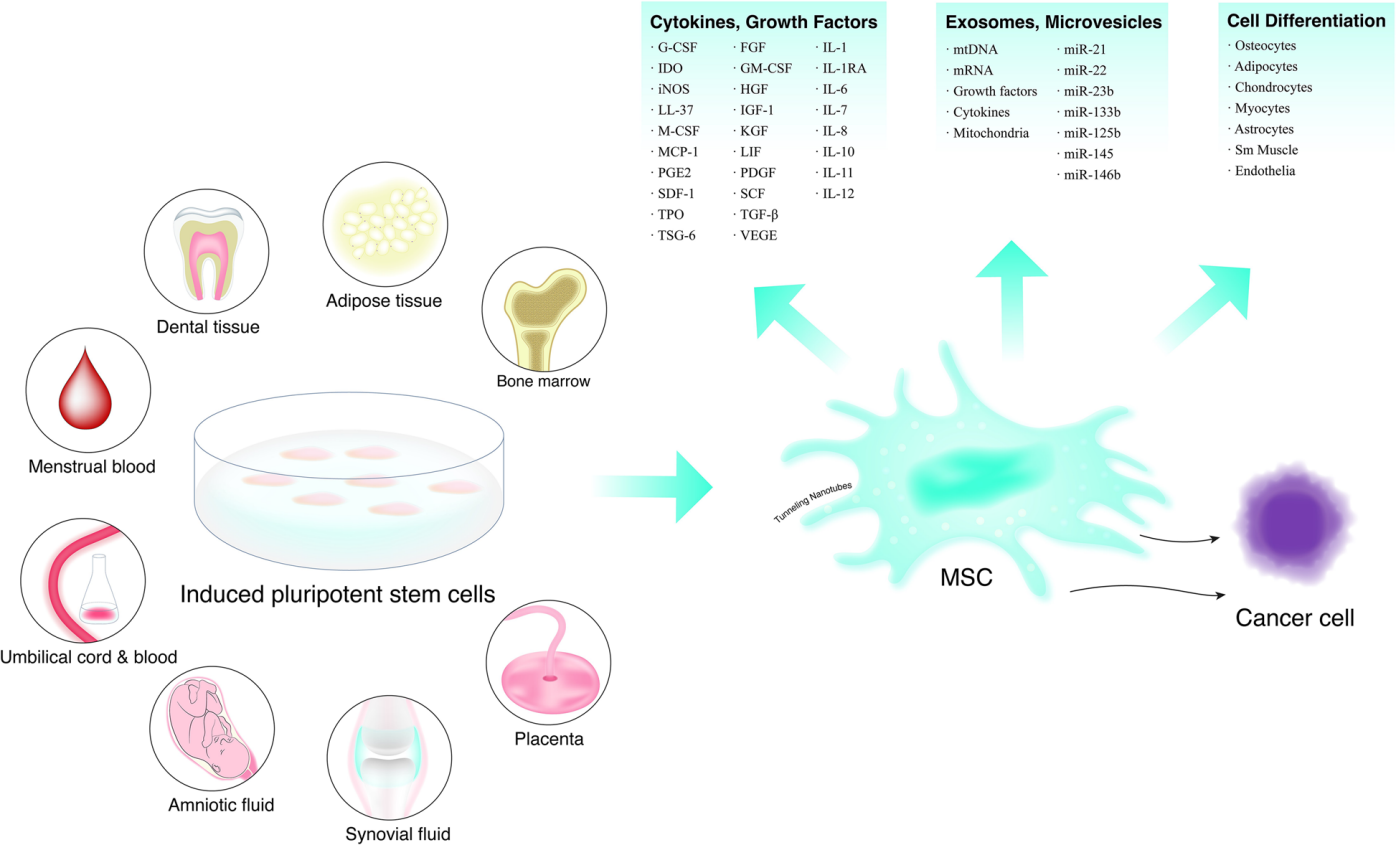
The origin of mesenchymal stem cell and its role. MSCs can come from bone marrow, placenta, umbilical cord, umbilical cord blood, adipose tissue, dental pulp, synovial fluid, amniotic fluid and induced pluripotent stem cells or embryonic stem cells. MSCs can differentiate into adipocyte, osteoblast, chondrocyte, and myocytes depending on their origins. The MSCs can also produce many growth factors and cytokines that regulate the immune responses, anti-inflammation, aid healing, alter host enhancing responses and serve as mature functional cells in tissue repair. The MSCs can also produce and release the microvesicles and exosomes encapsulate cytokines/growth factors/RNAs/miRNAs that have very similar function of MSCs. Notes: MSCs: Mesenchymal stem cells; G-CSF: Granulocyte colony stimulating factor; IDO: indoleamine 2, 3-dioxygenase; iNOS: Inducible nitric oxide synthase; LL-37: Leucine leucine-37; M-CSF: Granulocyte colony stimulating factor; MCP-1: Monocyte chemoattractant protein-1; PGE2: Prostaglandin E2; SDF-1: Stromal-derived factor-1; TPO: Thrombopoietin; TSG-6: Tumor necrosis factor inducible protein 6; TNF-stimulated gene-6; FGF: Fibroblast growth factor; GM-CSF: Granulocyte macrophage colony stimulating factor; HGF: Hepatocyte growth factor; IGF-1 insulin-like growth factor-1; KGF: Keratinocyte growth factor; LIF: Leukemia inhibitory factor; PDGF: Platelet-derived growth factor; SCF: Stem cell factor; TGF-β: transforming growth factor-βV; EGF: Vascular endothelial growth factor; IL: Interleukin; IL-1RA: Interleukin-1 receptor antagonist

MSCs, which exhibit differentiation and self-renewal capabilitis, are also called mesenchymal stromal cells or multipotent stromal cells and have been extensively investigated since their initial discovery. Researchers can obtain MSCs from many tissues and body fluids, such as placenta, umbilical cord, umbilical cord blood, Wharton’s jelly, bone marrow, dental pulp, adipose tissue, amniotic fluid and synovial fluid (Fig. 1) [17, 18]. In addition, MSCs can be obtained from embryonic stem cells or induced pluripotent stem cells [19]. Moreover, in terms of cluster of differentiation (CD), MSCs express CD73, CD90, and CD105 but not CD14, CD34, CD45, and human leucocyte antigen-DR (HLA-DR). Depending on their origins, MSCs can differentiate into chondrocytes, osteoblasts, myocytes and adipocytes [20].

**Fig. 2:**
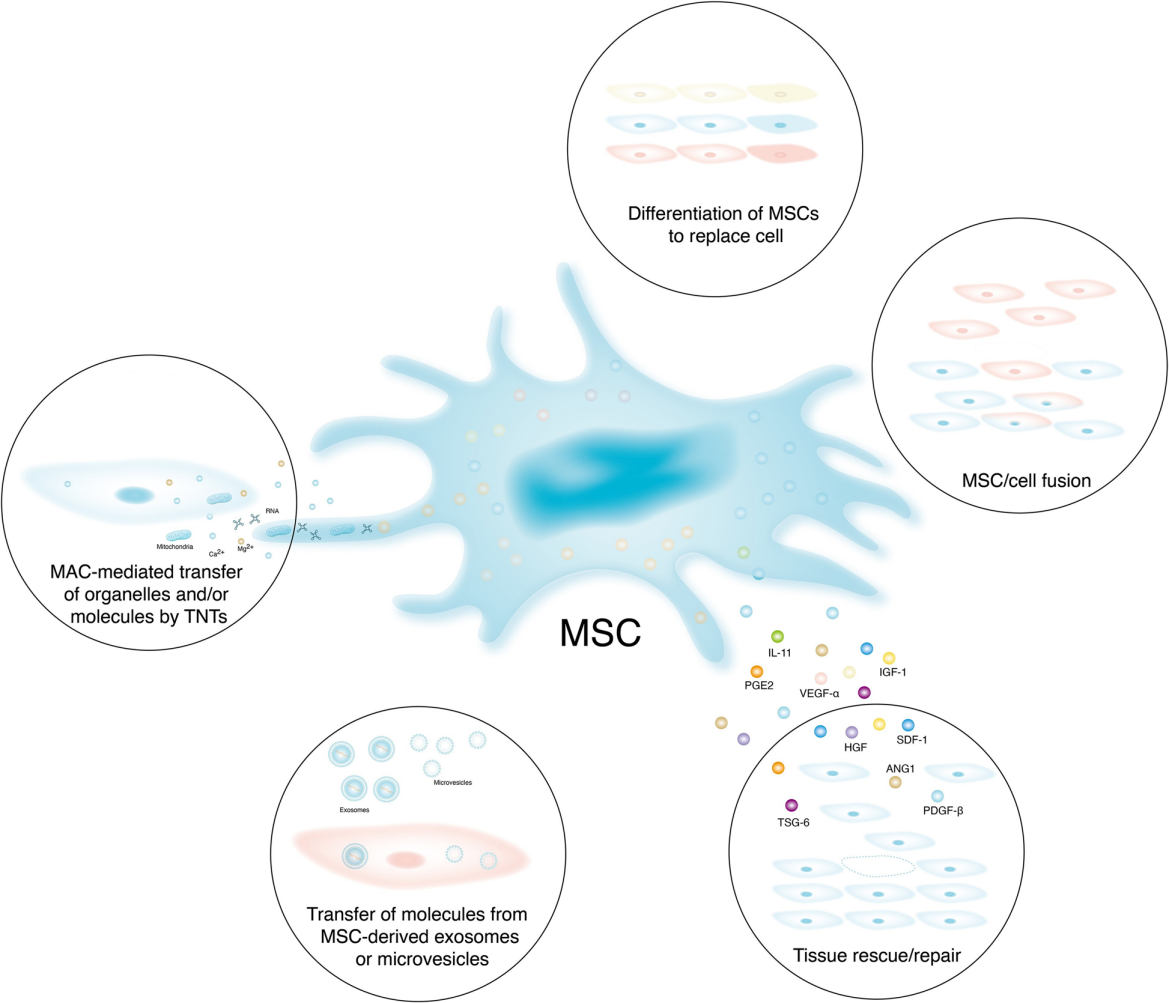
The role of MSCs rescue and/or repair tissues and injured cells by diverse mechanisms. **A** Differentiation into replacement cell types. **B** Rescue of damaged or dying cells through cell fusion. **C** Secretion of paracrine factors. **D **Transfer of organelles and/or molecules through tunneling nanotubes (TNTs). **E** MSC-mediated transfer of proteins/peptides, RNA, hormones, and/or chemicals by extracellular vesicles Notes: MSC: Mesenchymal stem cells; VEGF-α: Vascular endothelial growth factor-α; ANG1: angiopoietin-1; PDGF-β: Platelet-derived growth factor-β; IL-11: Interleukin-11; TSG-6: TNF-stimulated gene-6; PGE2: Prostaglandin E2; HGF: Hepatocyte growth factor; SDF-1: Stromal-derived factor-1; IGF-1: Insulin-like growth factor-1

MSCs have several simultaneous roles mediated by cell-to-cell interactions, secreted cytokines and growth factors, exosomes and cell differentiation (Fig. 1). The main roles of MSCs include (I) generating immune responses by the section of immunomodulatory proteins and through interactions with immune cells, such as lymphocytes, DCs, neutrophils, macrophages, mast cells and NK cells and through exosomes; (II) generating anti-inflammatory responses by the release of cytokines; (III) aiding healing by expressing growth factors; (IV) changing host-enhancing responses by endogenous repair cells; and (V) serving as mature functional cells in some tissues, such as bone [21–23]. Thus, through diverse mechanisms, MSCs have potent therapeutic effects in the context of various diseases (Fig. 2) [24, 25].

### Immunomodulatory function of MSCs

MSCs have immunomodulatory properties that depend on cell-to-cell contact and paracrine signaling. MSCs regulate several immune cells, such as T cells, B cells, DCs, NK cells and macrophages [26].

**Fig. 3:**
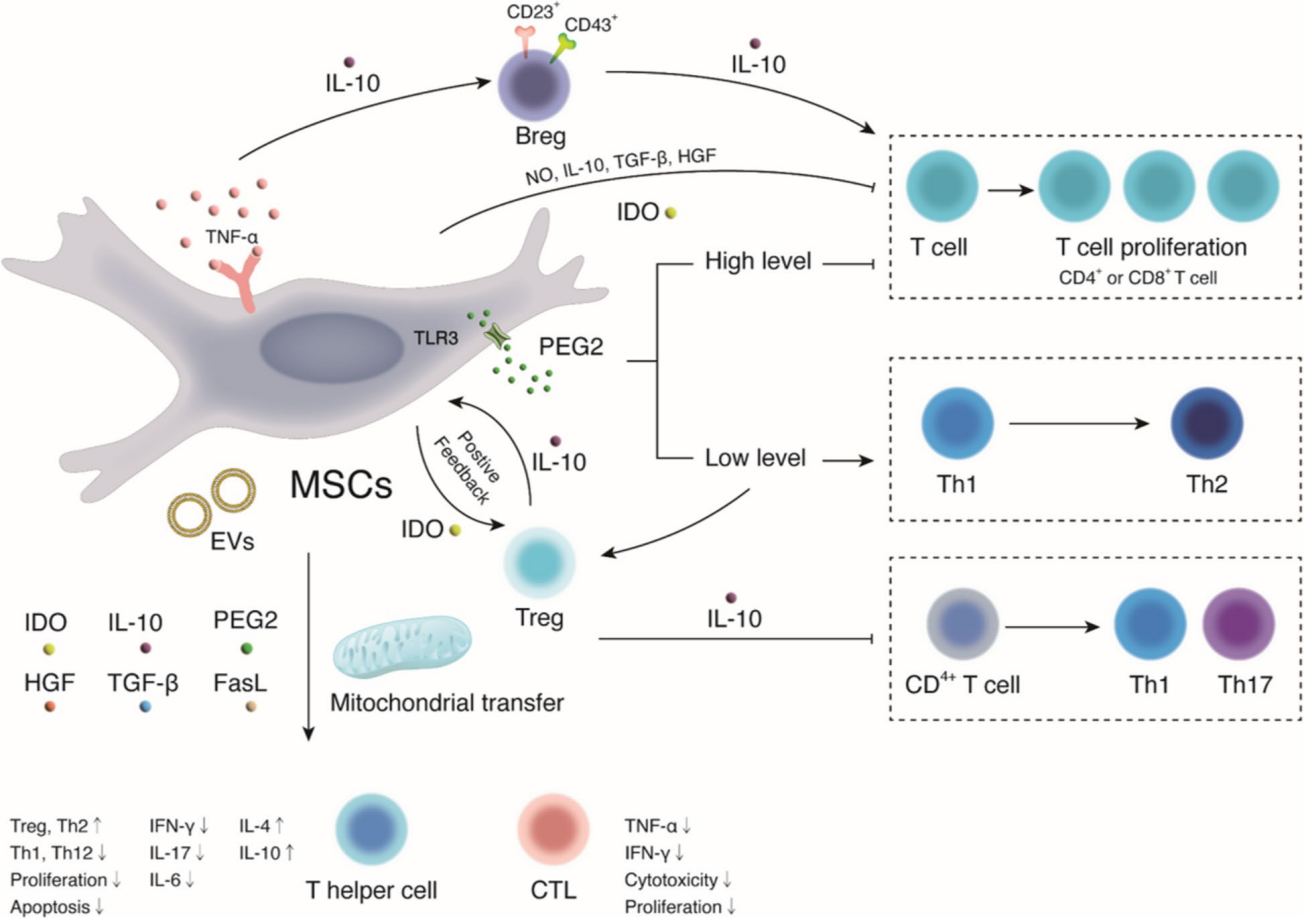
Immunoregulatory effects of MSCs on T cells in a contact-dependent manner or secrection of cytokines and growth factors or extracellular vesicles. MSCs inhibit the proliferation of T cells and activation of inflammatory Th1 and Th17 cells. In turn, MSCs induce immunosuppressive Tregs. Notes: MSCs: Mesenchymal stem cells; IL: Interleukin; Breg: Regulatory B cell; HGF: Hepatocyte growth factor; IFN-γ: Interferon-γ; NO: Nitric oxide; PGE2: Prostaglandin E2; Th: Helper T cell; Treg: Regulatory T cell; TGF-β: Transforming growth factor-β; TLR3: Toll-like receptor 3; EVs: Extracellular vesicles; FasL: Fas ligand; CTL: Cytotoxic T lymphocyte; TNF-α: Tumor necrosis factor-α; IDO: Indoleamine-2,3-dioxygenase; CD: Cluster of differentiation

The role of MSCs on T cells has two sides (Fig. 3). On the one hand, MSCs can inhibit the proliferation of T cells [27, 28]. MSCs are able to secrete nitric oxide to inhibit the cell cycle or apoptosis of T cells. Additionally, MSCs can increase the expression of p27kip1 and decrease the expression of cyclin D2 in T cells by secreting hepatocyte growth factor and transforming growth factor-β (TGF-β), which leads to cell cycle arrest in the G1 phase of T cells to inhibit their proliferation. MSCs can also secrete prostaglandin E2 (PGE2), tumor necrosis factor-α (TNF-α) and interferon-γ (IFN-γ) to inhibit the proliferation and induce the apoptosis of T cells [29]. A low concentration of tryptophan can induce a decrease in T cell levels [30]. However, MSCs can modulate T-cell activation, differentiation, and effector function. Cytokines secreted by MSCs suppress the activation of naive T cells and change the differentiation process of T-cell subsets. Cytokines can increase the production of interleukin-10 (IL-10) and decrease the production of TNF-α and IL-12 by inhibiting proinflammatory T cells and inducing the production of regulatory T cells (Tregs) [31, 32]. Nevertheless, at a certain concentration, IL-10 is able to suppress the activation of CD4^+^ T cells to Th1 and Th17 and induce the secretion of soluble human leukocyte antigen-G5 and the production of Tregs [33]. MSCs are able to express intercellular adhesion molecule-1 and vascular cell adhesion molecule-1 to increase the adhesion of MSCs and T cells and exert immunosuppressive effects on T cells [34]. The apoptosis of inflammatory T cells can be induced by the Fas/FasL signaling pathway [35]. The activation of Jagged-1/Notch-1 signaling can induce the differentiation of CD4^+^ T cells into Tregs [36]. Through both cell contact and paracrine effects, the differentiation of Th17 cells can be effectively repressed by the activation of programmed death-1/programmed death-1 ligand (PD-1/PD-L1) signaling [37].

**Fig. 4:**
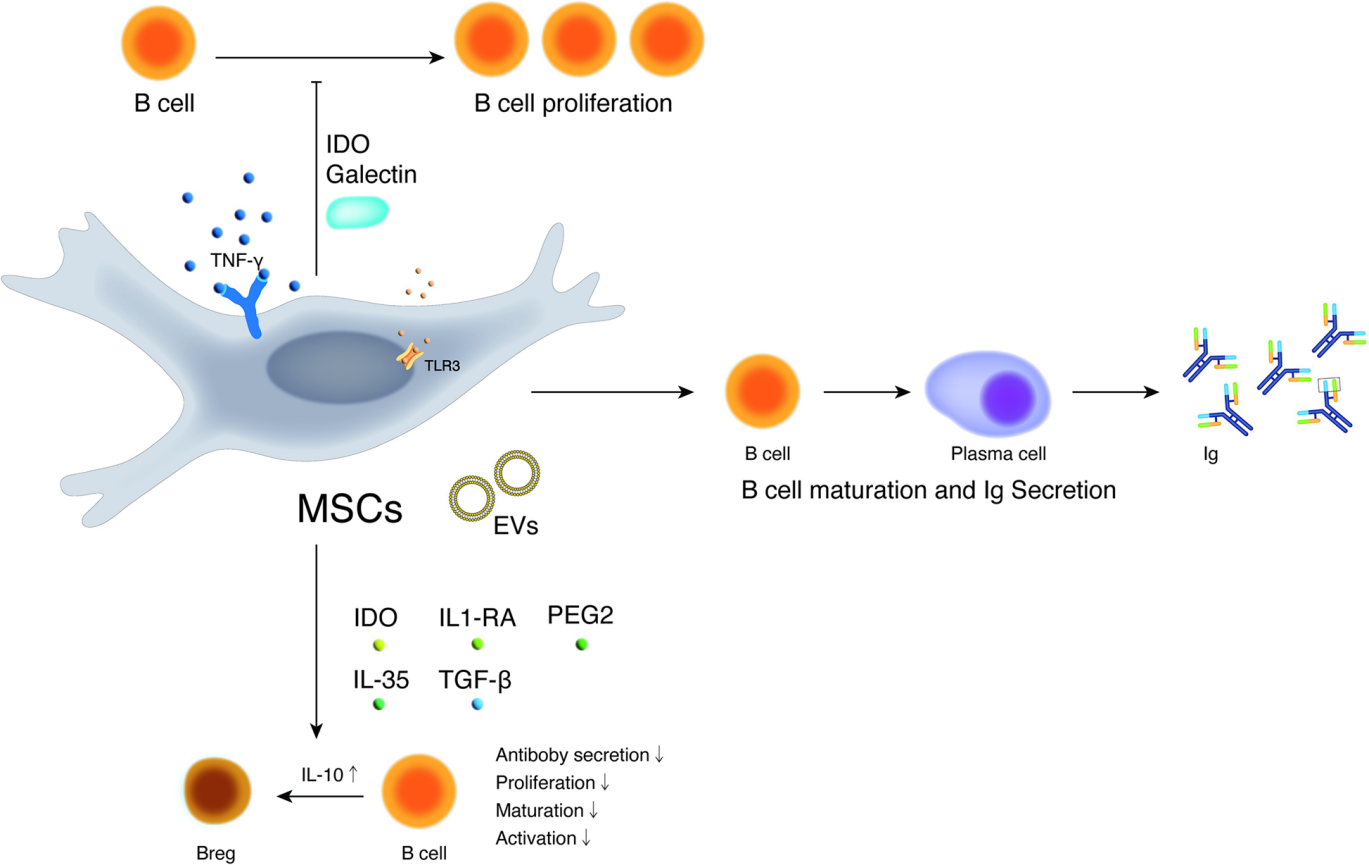
Immunoregulatory effects of MSCs on B cells in a contact-dependent manner or secrection of cytokines and growth factors or extracellular vesicles. MSCs decrease B-cell activation and proliferation and attenuate immunoglobulin production. Notes: MSCs: Mesenchymal stem cells; IL: Interleukin; Ig: Immune globulin; EVs: Extracellular vesicles; IDO: Indoleamine-2, 3-dioxygenase; TNF-γ: tumor necrosis factor-γ; PGE2: Prostaglandin E2; TGF-β: Transforming growth factor-β; TLR3: Toll-like receptor 3; IL-1RA: Interleukin-1 receptor antagonist

Fewer studies have investigated the effects of MSCs on the immunomodulation of B cells than on that of T cells (Fig. 4). The proliferation and differentiation of B cells are inhibited by MSCs through modifying the phosphorylation pattern of p38 mitogen-activated protein kinase and serine/threonine kinase [38]. Nevertheless, the immunomodulatory activity of B cells can be significantly enhanced through the upregulation of IL-10 on MSCs. MSCs can induce the production of a population of CD23^+^CD43^+^ regulatory B cells (Bregs) and inhibit the secretion of proinflammatory cytokines and the proliferation of T cells through the IL-10-dependent pathway [39, 40]. MSCs suppress the secretion of immune globulin from plasma cells by inhibiting the expression of activator of transcription 3 and transcription factor signal transducer and inducing the expression of paired Box 5 [41]. MSCs activated by IFN-γ inhibit the proliferation and maturation of B cells by activating PD-1/PD-L1 signaling [42].

**Fig. 5:**
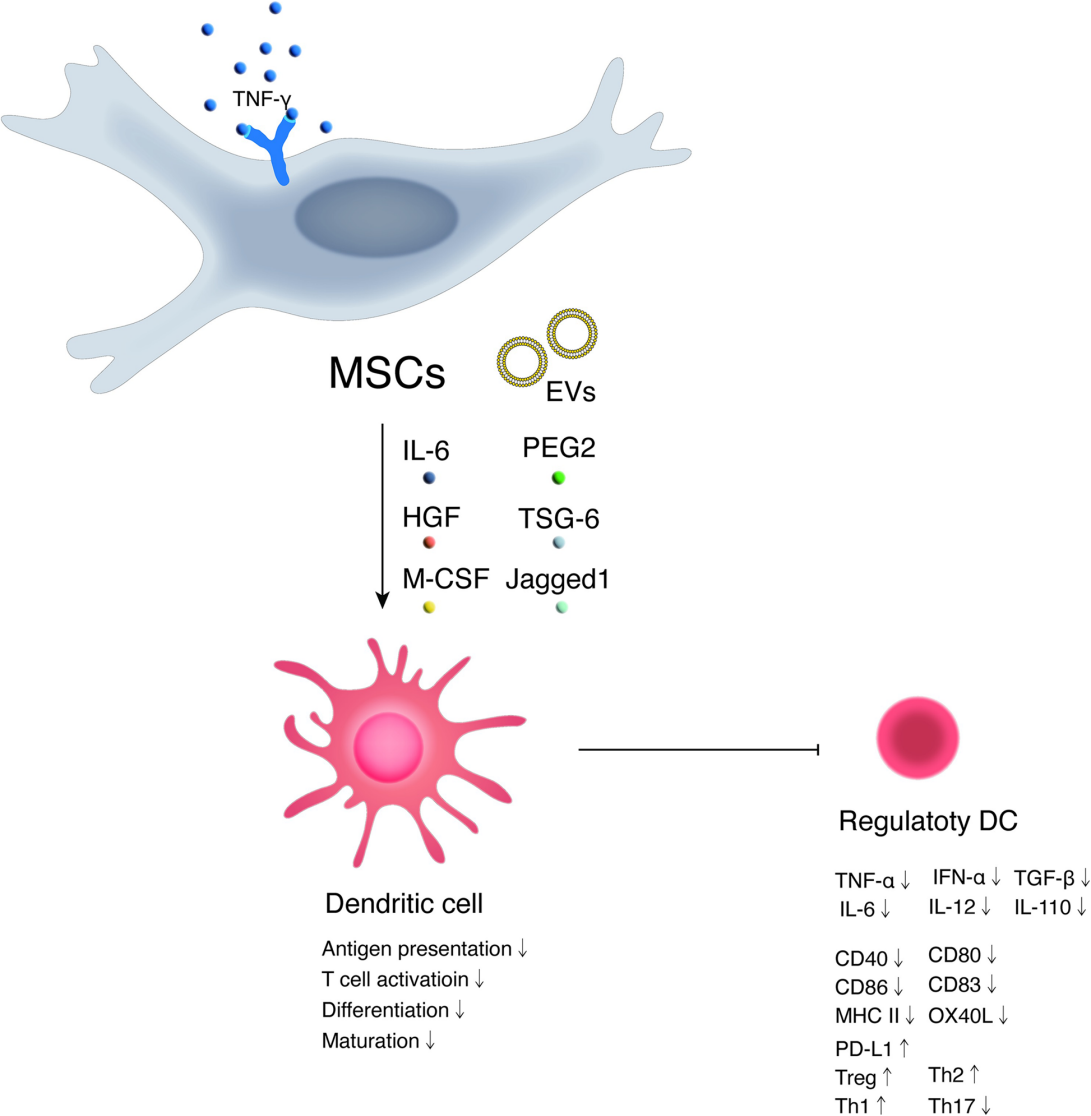
Immunoregulatory effects of MSCs on dendritic cell in a contact-dependent manner or secrection of cytokines and growth factors or extracellular vesicles. MSCs mainly prevent monocyte differentiation to DCs. Notes: MSCs: Mesenchymal stem cells; DC: dendritic cell; IL: Interleukin; PD-L1: Programmed death-1 ligand; HGF: Hepatocyte growth factor; TNF-γ: tumor necrosis factor-γ; PGE2: Prostaglandin E2; Th: Helper T cell; Treg: Regulatory T cell; TGF-β: Transforming growth factor-β; EVs: Extracellular vesicles; TNF-α: Tumor necrosis factor-α; TSG-6: TNF-stimulated gene-6; M-CSF: Macrophage colony stimulating factor; MHCII: Major histocom-patibility complex II; IFN-α: Interferon-α

DCs, which exhibit remarkable phenotypic and functional plasticity have the potential to regulate the T-cell response (Fig. 5). However, normally, MSCs prevent monocyte differentiation into DCs [43–45]. Human umbilical cord blood-derived MSCs can reverse the production of mature DCs that stimulate the T-cell response via the downregulation of MHC-II [46]. In addition, the expression of costimulatory molecules such as CD80 and CD86 is also suppressed. MSCs generated from induced pluripotent stem cells inhibit the differentiation of DCs by both IL-10 and direct cell contact. These MSCs are able to increase their phagocytic ability and inhibit the proliferation of lymphocytes to regulate their function; nevertheless, their activities do not affect the maturation of DCs [47].

**Fig. 6:**
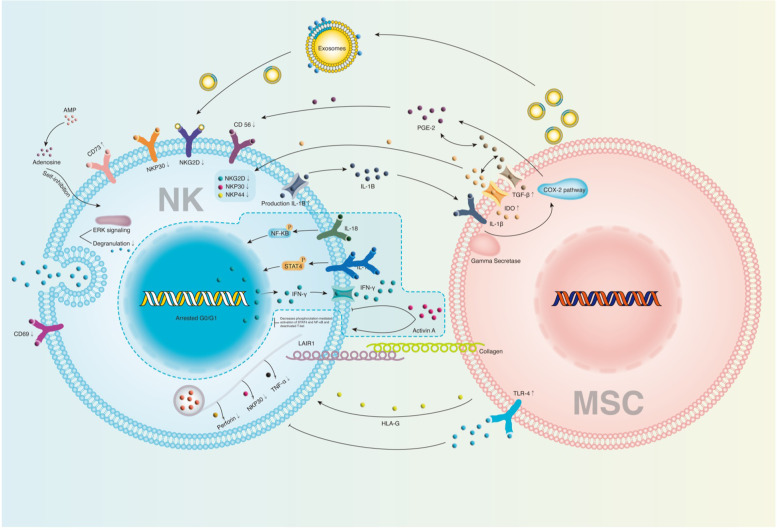
Immunoregulatory effects of MSCs on NK cells in a contact-dependent manner or secrection of cytokines and growth factors or extracellular vesicles. MSCs mainly inhibit the proliferation of NK cells. Notes: MSCs: Mesenchymal stem cells; NK cells: Nature killer cells; IL: Interleukin; HLA-G: human leukocyte antigen-G; IFN-γ: Interferon-γ; PGE2: Prostaglandin E2; TGF-β: Transforming growth factor-β; TLR4: Toll-like receptor4; TNF-α: Tumor necrosis factor-α; IL-1β: Interleukin-1β; AMP: Adenosine monophosphate; LAIR1: Leukocyte-associated immunoglobulin-like receptor 1; NFKB: Nuclear factor kappa-light-chain-enhancer of the activated B cell; COX-2: Cyclooxygenase-2; ERK: Extracellular signal-regulated kinase; NKP: Nature killer cell protein; NKG2D: Natural killer group 2 member D; CD: Cluster of differentiation; IDO: Indoleamine-2,3-dioxygenase

Regarding NK cells, MSCs can regulate their function (Fig. 6) and reduce the secretion of IFN-γ by purified IL-2-stimulated NK cells [48, 49]. In addition, MSCs can inhibit the proliferation of NK cells in an IFN-γ-dependent manner [50]. MSCs can suppress the cytotoxicity of NK cells and IFN-γ secretion by releasing factors such as indoleamine 2,3-dioxygenase (IDO) and PGE2 [51–53]. MSCs obtained from induced pluripotent stem cells can decrease the cytotoxicity of NK cells by regulating ERK1/2 signaling and the expression of activation markers [54].

**Fig. 7:**
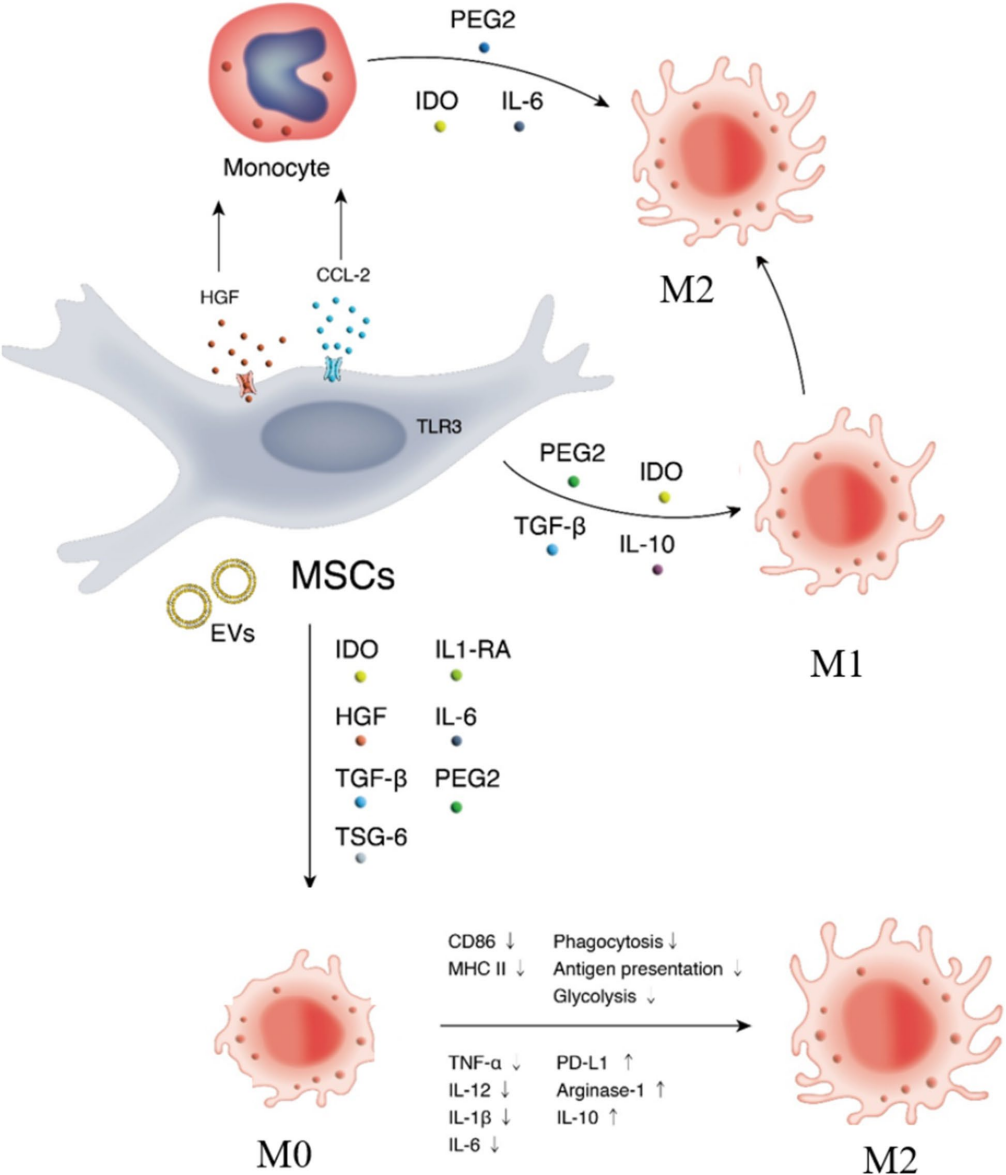
Immunoregulatory effects of MSCs on macrophage in a contact-dependent manner or secrection of cytokines and growth factors or extracellular vesicles. MSCs suppress macrophage migration and regulate their polarization. Notes: MSCs: Mesenchymal stem cells; EVs: Extracellular vesicles; PD-L1: Programmed death-1 ligand; HGF: Hepatocyte growth factor; IDO: Indoleamine-2,3-dioxygenase; M: Macrophages; PGE2: Prostaglandin E2; TGF-β: Transforming growth factor-β; TNF-α: Tumor necrosis factor-α; TSG-6: TNF-stimulated gene-6; IL: Interleukin; C–C motif chemokine ligand 2; IL-1RA: Interleukin-1 receptor antagonist

Regarding macrophages, MSCs have the ability to produce various chemokines, signaling molecules and growth factors to affect their proliferation, maturation, polarization, and migration (Fig. 7) [55, 56]. The coculture of MSCs with macrophages can induce the polarization of the latter from the M1 phenotype to the M2 phenotype, which could inhibit antigen-presenting cell infiltration and enhance the activity of macrophages [57, 58]. The mechanism by which MSCs affect the polarization of macrophages is mediated by direct cell contact and cytokines, such as IL-1α, IL-6, IL-10, TGF-β, IDO and PGE2 [59–61].

Altogether, these data suggest that MSCs can regulate several immune cells. However, the different tissue-derived MSCs have different immunomodulatory abilities and proliferation properties in vitro. Thus, comparative studies of MSCs of different origins are vital to identify the most suitable cells for immunomodulation for clinical use in the treatment of different diseases.

### MSCs as a source of exosomes

Extracellular vesicles (EVs) can be released by all living cells and consist of a very diverse group of cell-derived, lipid bilayer-enclosed vesicles, including shedding vesicles, apoptotic bodies and exosomes. Shedding vesicles are also called ectosomes, ectovesicles or microvesicles, and they are directly formed by outward budding of the plasma membrane. Apoptotic bodies are released by dying apoptotic cells. Exosomes are secreted by the fusion of multivesicular bodies (MVBs) with the plasma membrane. MVBs are formed by inward budding from the external membrane of late endosomes and successive pinching off of budding vesicles into the lumenal space of late endosomes [62]. MVBs can form intralumenal vesicles, which are called exosomes when they are released into the extracellular medium. The three types of EVs have different sizes, shapes, origins and markers (Table 1).

**Table 1:** Characteristics of extracellular vesicles

	Apoptotic bodies	Microvesicles	Exosomes
Origin	Cells undergo apoptosis	Cell’s plasma membrane	Endosomal compartment
Shape	Irregular	Irregular	Regular
Size	50–5000 nM	100–1000 nM	30–150 nM
Markers	Intact organelles, chromatin,histones	Cytoskeletal proteins, heat shock proteins, integrins	CD9, CD63, CD81, Alix, TSG100

**Fig. 8:**
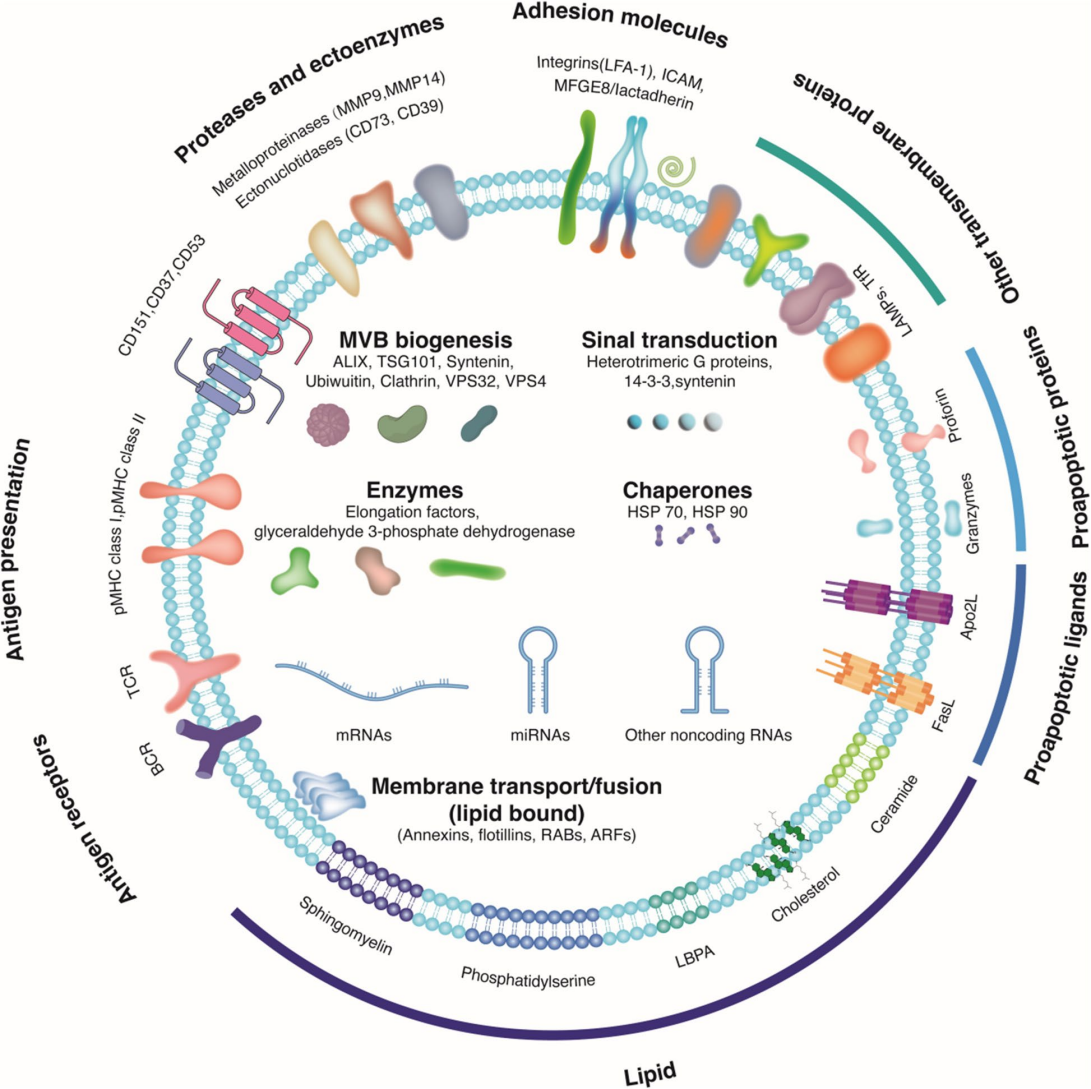
Exosome structure and molecular composition. Exosomes are surrounded by a phospho-lipid bilayer and contain nucleic acids and lipids, proteins. Exosomal proteins include annexins, important for transport; integrins and tetraspanins important for cell targeting and binding, and TSG101and Alix, involved in exosomal biogenesis from endosomes. Notes: MHC: Major histocom-patibility complex; HSP: Heat shock protein; TSG101: Ttumor susceptibility gene 101; TCR: T cell receptor; BCR: B cell receptor; CD73: Cluster of differentiation 36; CD39: Cluster of differentiation 39; MMP9: Matrix metalloproteinase-9; MMP14: Matrix metalloproteinase-14; MFGE8: milk fat globuleEGF factorVIII; ICAM: Intercellular adhesion molecule; LFA-1: Lymphocyte function-associated antigen-1; VPS32: Vacuolar protein sorting-associated protein 32; FasL: Fas ligand; LBPA: Lysobisphosphatidic acid; ARFs: Auxin response factors; RABs: Rab family of proteins; ALIX: ALG-2 interacting protein X; mRNA: Messenger nucleic acid; miRNA: Microribonucleic acid

Exosomes have receptors, transmembrane proteins, transcription factors, enzymes, extracellular matrix proteins, lipids and nucleic acids (mRNA, DNA, and miRNA) within their lumens and on their surfaces (Fig. 8) [63]. Exosomes can transfer their cargo (proteins, lipids, miRNAs) between cells and may also trigger certain cues in recipient or target cells [64–66]. Therefore, exosomes affect the physiology of neighboring target cells in diverse ways by triggering cell signaling on through cell surface receptors and then generating new functions after the acquisition of enzymes, novel receptors or genetic materials in the target cells [63, 67].

MSC-derived exosomes can deliver various RNAs, DNAs, proteins and lipids, which can promote MSCs migration, tissue repair, and immunomodulation and promote certain functions in target cells [15, 68, 69]. Therefore, MSC-derived exosomes are considered a promising alternative therapy for various diseases [70–72]. MSCs are an abundant resource, and the characteristics and functionality of MSC-derived exosomes depend on their origins (Table 2) [73–78]. However, most of the results of previous studies have not been directly obtained from comparative studies because the methods used for the isolation, characterization, and efficacy evaluation of exosomes are not comparable. This discrepancy still a prominent challenge that is caused by variations among different donors or MSCs preparation methods [79, 80].

**Table 2:** Role of MSCs derived exosomes from different sources

Diseases	MSCs Origin	Response	Ref
Alzheimer’s disease	Human adipose tissue Human BM	Adipose MSCs derived exosomes have superior effects compared to BM-MSCs derived exosomes Decreased Aβ peptide in the N2a cells	[73]
Neurodegenerative disease	Human menstrual tissue Human BM	Promoted neurite outgrowth in cortical and sensory neurons	[75]
	Human chorion Human umbilical cord	No effect	
Osteoarthritis (OA)	Human iPSCs Human SM Human BM	Attenuated OA in a murine model Stimulated chondrocyte migration and proliferation iMSCs derived exosomes exert superior therapeutic effects compared to SM-MSCs derived exosomes	[76]
Glioblastoma	Human BM Human Wharton’s jelly	Decreased the proliferation of U87MG cell Induced apoptosis in the U87MG cells	[74]
	Human adipose tissue	Increased the proliferation of U87MG cell No apoptotic effect	

Notes: *MSCs* Mesenchymal stem cells, *BM* Bone marrow, *AF* Amniotic fluid, *ASC* Adipose stem cell, *BM* Bone marrow, *iMSC* Induced pluripotent stem cell-derived MSCs, *OA* Osteoarthritis, *SM* Synovial membrane, *iPSCs* Induced pluripotent stem cells, *Ref*. Reference

In conclusion, due to the differences in the origin of MSCs, MSC-derived exosomes present different properties and efficacies. Nevertheless, several studies have widely explored the use of MSC-derived exosomes in the treatment of various diseases [81–84].

### Immunomodulatory functions of MSC-derived exosomes

**Fig. 9:**
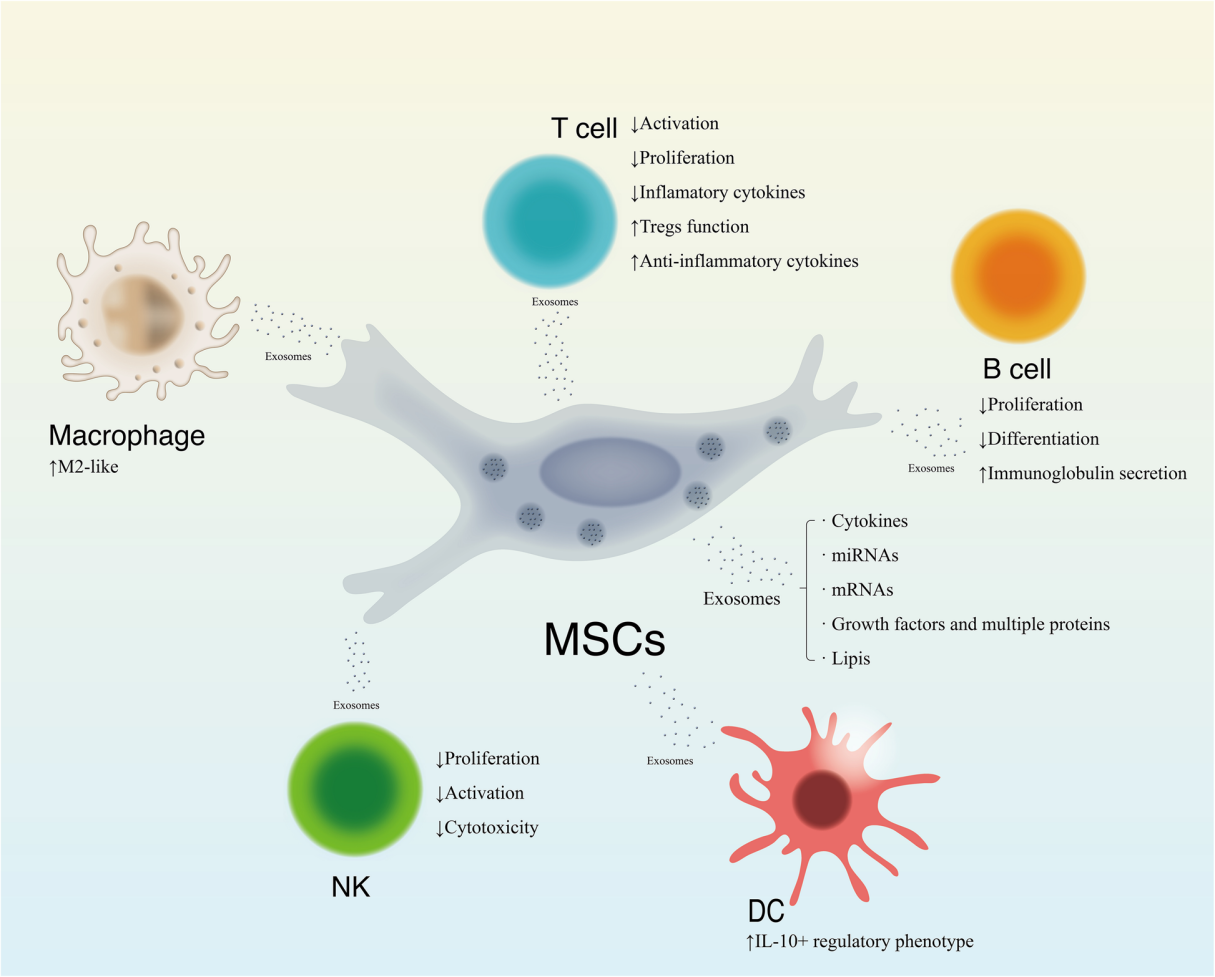
Immunoregulatory effects of MSCs derive exosomes on immune cells in a contact-dependent manner. The role and mechanism of immunological tolerance of MSC-EVs on immune cells. The EVs derived from MSC play immunological tolerant role on the innate and adaptive immune responses including extensive immune cells. MSC-EVs could suppress the activation and proliferation of T cell and reduce production of inflammatory cytokines, while improve the Treg function and anti-inflammatory cytokines generation. Similarly, MSC-EVs play the suppressive role on the proliferation, differentiation, and immunoglobulin secretion of B cell. Considering the innate immune cells, MCS-EVs induce IL-10-expressing regulatory phenotype of DCs and inhibit the co-stimulatory molecules of monocytes. The macrophage would adopt anti-inflammatory M2 phenotype after MSC-EVs stimulation. Notes: MSCs: Mesenchymal stem cells; NK: Nature killing cells; DC: Dendritic cell; IL: Interleukin; mRNAs: Messenger nucleic acids; miRNA: Microribonucleic acids. EVs: Extracellular vesicles

MSC-derived exosomes have immunomodulatory roles in T cells, B cells, DCs, macrophages and NK cells mediated by their delivery of various RNAs, DNAs, proteins and lipids (Fig. 9). In addition, the biological functions of MSC-derived exosomes are similar to those of their cells of origin, but exosomes have lower immunogenicity and higher stability [85]. A variety of studies have widely reported the immunomodulatory abilities of MSC-derived exosomes [22].

The exosomes that target T cells and are derived from MSCs can induce the conversion of Th1 cells into Th2 cells, increasing the level of Tregs, reduce the differentiation of Th17 cells, induce the apoptosis of T cells, and promote the infiltration and proliferation of proinflammatory T cells via cytokines and growth factors [86–88]. Regarding B cells, exosomes derived from MSCs can reduce their proliferation [89]. MSC-derived exosomes suppress the maturation of bone marrow DCs by decreasing the expression of surface markers on DCs by decreasing IL-6 release while augmenting IL-10 and TGF-β release, and the proliferation of lymphocytes is reduced in the presence of DCs [90]. The main role of MSC-derived exosomes in the immunomodulation of macrophages involves inhibiting the recruitment of macrophages and inducing M1/M2 polarization through downregulation or upregulation of cytokines via exosomal miR-223, miR-181c, miR-182, let-7b, let-7, MT2A, and STAT3 in exosomes [91–93].

The exosomes originating from the MSCs of the fetal liver have been found to lead to the inhibition of the activation, cytotoxicity and proliferation of NK cells [94]. This function is induced by downstream TGFβ/Smad2/3 signaling in NK cells via the latency-associated peptide TGF-β and thrombospondin 1 in exosomes. MSC-derived exosomes increase the proportion of DCs (CD11b^+^/CD11c^+^) in spleen and tumor tissues in clear renal cell carcinoma [95].

In conclusion, MSC-derived exosomes have roles that are similar to their origin MSCs. However, MSC-derived exosomes obtained from different tissues have different immunomodulatory abilities. Thus, comparative studies of different types of MSC-derived exosomes are vital to understand their immunomodulatory characteristics.

### The role of MSCs in CAR-related products

#### The role of CAR-related products

**Fig. 10:**
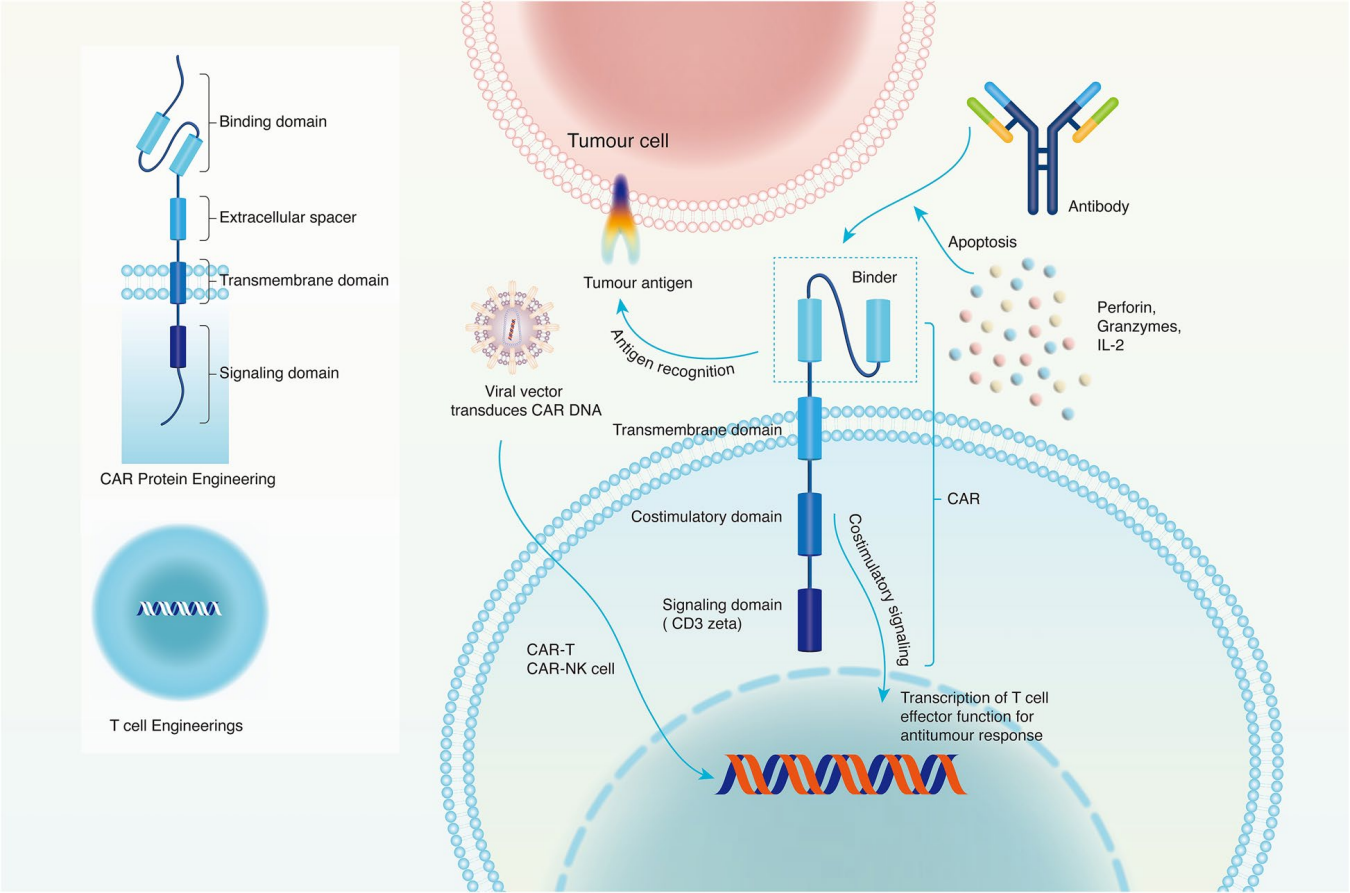
The structure and construction of CAR-T and the mechanism of anticancer. The T/NK cell can be engineered to express CAR with its binder from monoclonal antibodies. The CAR consist of the co-stimulatory domain and signalling domain (CD3zeta). A viral vector is used to transfer the DNA that codes for CAR into the nucleus of the immune cell. The signal is amplified and transferred to the nucleus after the CAR receptor recognizes the tumor antigen. Then, the T/NK cell may proliferate and secrete cytokines, perforins to initiate a series of antitumor responses. Notes: CAR: Chimeric antigen receptor; IL-2: Interleukin 2

The CAR comprises the extracellular tumor-antigen receptor and intracellular signal transduction domain. The former, which includes the antigen-binding site of monoclonal antibodies, specifically recognizes TAAs on the cell-surface membrane of tumor cells. The latter, which comprises the combination of a natural TCR complex and costimulatory molecules, stimulates the proliferation and function of engineered cells (Fig. 10) [96]. The design of a CAR used for the treatment of tumors depends on specific TAAs, while costimulatory (4-1BB or CD28 for CAR-T cells) and signaling domains (CD3zeta for CAR-T cells) rely on the carrier of immune cells [97]. Several generations of CARs have been produced according to the intracellular signal transduction domain.

Several studies have reported vital achievements of CAR-T-cell regimens used in the treatment of hematological malignancies [98–101]. However, many challenges need to be resolved, including poor T-cell persistence, T-cell dysfunction or exhaustion, relapse of disease, severe CRS and ICANS, tumor lysis syndrome, off-tumor on-target toxicity, low efficacy against solid tumors and immunosuppression by the tumor microenvironment [102–107].

Although there are no data on CAR-B cells, the presence of CARs in leukemia B cells has been detected in a patient treated with CAR-T cells [108]. CAR-B cells may be used to deliver monoclonal antibodies and as a novel platform for prophylactic vaccines and autoimmune disease [109].

CAR-NK cells, as an alternative candidate for retargeting cancer, have demonstrated powerful cytotoxic effects on tumor cells, clinical safety and unique recognition mechanisms [110]. However, many challenges remain, such as low persistence, low efficacy of transport to the required tumor site, and low lentivirus transduction efficiency [111].

CAR-Ms can specifically clear the tumors through antigen-specific phagocytosis in vitro [112]. Notably, the infusion of human CAR-Ms can extend overall survival and reduce tumor burden. CAR-Ms can convert M2 macrophages to the M1 macrophages, express pro-inflammatory cytokines and chemokines, upregulates genes involved in antigen presentation machinery, produce activation and maturation markers in immature human DCs, and can recruit both resting and activated human T cells. CAR-Ms can also significantly induce increased proliferation and killing of T cells. The CAR-Ms showed a good effect on tumors [113, 114]. CAR-Ms can be used as an alternative approach for tumor therapy with high antitumor activity. CAR-Ms act not only as phagocytic machinery but also as antigen presenters, immune stimulators and modifiers to promote anticancer immunity [1]. However, some other obstacles need to be overcome for the use of CAR-Ms, such as, the differentiation and retention of the M1 phenotype, and the clinical assessment of the safety and effectiveness of CAR-Ms [1].

Recent research has studied the role of CAR-DCs in anticancer therapy [115]. In one study, bone marrow CD34^+^ progenitors and T cells were sorted. Cells were transduced with an anti-CD33 41BBz CAR lentivector (pCCL-HP67.6–4-1BB-CD3z). The transduced CD34^+^ cells were induced to differentiate into DC (CAR-DCs) in vitro by incubating the cells with Flt3L/GM-CSF/IL-4 and acute myeloid leukemia cell lysate. Kasumi-1 cells were cocultured with CAR-T-cells with or without CAR-DCs. Tuciferase-GFP tagged Kasumi-1 cells were used to infect NSG mice, followed by injection of CAR-T cells with or without CAR-DCs. The results showed that CAR-DCs can differentiate into the intratumoral DC subset and improve the cytotoxicity of infused CD33-CAR T cells with higher cytokine production and better survival in mouse xenografts [115].

#### Role of CAR-related product-derived exosomes

In immunotherapy, exosomes that originated from CAR-T cells have considerable antitumor properties. The presence of the CAR molecule on exosomes is crucial for CAR-T-cell-derived exosomes to specifically induce tumor cell death [7, 71]. Recent studies have shown that CARs are present in exosomes and have antitumor effects and low toxicity (Table 3) [116–119]. Exosomes from CAR-T cells with EGFR and HER2-specific CARs can specifically induce the apoptosis of tumor cells expressing the antigens recognized by CAR on the cell surface. The exosomes that bind to and penetrated specific target cells are also vital [120]. Several CAR cells do not express apoptotic molecules, such as Apo2L, perforin, FasL and granzymes. The exosomes from CAR-T cells with signal recognition particle 7SL1 (RN7SL1, a noncoding RNA that activates interferon-IFN-stimulated genes) can orchestrate endogenous immune activation to improve responses against the tumor [121]. These exosomes also transfer RN7SL1 to myeloid cells, DCs and T cells but not to tumor cells, which improves the immunostimulatory role of DCs and myeloid cells and effectively activates the function of endogenous CD8^+^ T cells against the tumor.

**Table 3:** Compare of CAR-T cells and CAR-T cells derived EV

	CAR-T cells	CAR-T cell derived EV
Cytokine releasing syndrome	+ +	* − *
Neurotoxicity	+ +	* − *
Cross the blood barrier	* − *	+ +
Efficiency against solid tumors	+ /* − *	+ +
Immunosuppression by tumoral PD-L1	+	* − *
Immunological memory	+ ^1^	(?)^2^

Notes: *CAR-T cells* Chimeric antigen receptor T cells, *EV* Extracellular vesicles, *PD-L1* programmed death-1 ligand,

^1^Depends on the use of central memory or effector memory CAR T cells

^2^Not formally established

As a cell-free immunotherapy, the substantial advantages of CAR-T-cell-derived exosomes include their independence from the CAR-T-cell lifespan, division and stability and the low risk of collateral toxicity compared to CAR-T cells. Moreover, exosomes can be distributed via the blood circulation and other body fluids. In addition, exosomes can cross specific biological barriers, such as the blood–brain barrier [122].

#### Effect of MSCs on CAR-related products

MSCs regulate T cells, in two ways, as described above [123]. Recent data have shown that MSCs can regulate the function of CAR-T cells (Table 4). Although bone marrow MSCs (BM-MSCs) from multiple myeloma (MM) can significantly protect MM cells from lysis by lower affinity, moderately lytic BCMA-, CD38-, and CD138-specific CAR-T cells only in a cell-to-cell contact-dependent manner, MM cells can be killed by high-affinity, strongly lytic BCMA- and CD38-CAR-T cells [124]. BM-MSCs did not reduce the secretion of IFNγ and granzyme B in UM9 cells or patient MM cells by BCMAC^11D5.3^-CAR-T cells and BBz-CD38^B1^-CAR-T cells. Instead, the secretion of IFNγ and granzyme B increased in the MM cells of patients. The secretion of IFNγ was reduced in two primary MM samples in the presence of BM-MSCs for CD138-CAR T cells. However, BM-MSCs did not reduce granzyme B secretion. All of these results show that BM-MSCs partially inhibit CAR-T cells.

**Table 4:** Effect of MSCs on CAR related product

MSCs origin	MSCs modified	CAR type	Tumor cells	Effect on CAR response	Ref
BM of MM patients	/	BCMA.CAR-T cells CD38.CAR-T cells CD138.CAR-T cells	MM	Protect the lysis by low affinity, moderately lystic CAR; More killed by high affinity, strongly lytic CAR	[124]
BM of B-ALL patients. BM of Health donor	/	CD19.CAR-T cells	B-ALL	Inhibiting the growth of B-ALL	[125]
BM of Health donor	CAd.MSCs	HER2.CAR-T	A549、H1650 lung cancer cell lines	Increase the cytotoxicity	[13]
BM of Health donor	IL-2.IL7.MSCs	CEA.CAR-T cells	LS174T colorectal cancer cells	Both non-modified and modified MSCs can improve the cytotoxicity. The modified MSCs have higher cytotoxicity	[16]
BM of MM patients	/	CD38.CAR-T cells	RPMI-8226 and MM1.s MM cell lines	Increase the lysis	[129]

Notes: *MSCs* Mesenchymal stem cells, *BM* Bone marrow, *CAR* Chimeric antigen receptor, *CAR-T cells* Chimeric antigen receptor T cells, *MM* Multiple myeloma, *B-ALL* B cells acute lymphoblastic leukemia.CAd: combinatorial adenoviruses vector. Ref. Reference

A recent study showed that BM-MSCs from both patients with B-cell acute lymphoblastic leukemia (B-ALL) and healthy donors strongly inhibit the T-cell response but not CD19. CAR-T-cell activity [125]. The growth of CD19-positive tumor cells was controlled in vivo by CD19. CAR-T cells, regardless of the presence or absence of MSCs in healthy donors and B-ALL patients, the levels of IFN-γ, IL-2 and TNF-α were also similar in culture supernatants.

MSCs can contain oncolytic immunotherapy agents with engineered adenoviruses (OAd) together with a helper-dependent Ad (HDAd; combinatorial Ad vector [CAd]) expressing PD-L1 blockers and IL-12 can be delivered and produce a functional virus to infect and lyse lung tumor cells. Moreover, it stimulates the antitumor activity of CAR-T cells by releasing PD-L1 blockers and IL-12. This method also increases the overall numbers of human T cells in vivo compared to treatment with only CAR-T-cells and enhances the secretion of polyfunctional cytokines [13].

IL-7 can sustain the memory cell function of T cells [126]. IL-12 protects the Th1 response and prevents Th2 polarization of T cells [127]. IL-12 can also eliminate cancer cells resistant to CAR-T cells by activating an innate immune response [128]. The BM-MSCs of healthy donors can increase the amplification of CAR-T cells when coincubated with CAR-T cells, inhibit the activation of induced cell death (AICD) in higher numbers, and sustain and enhance the antitumor activity of CAR-T cells against colorectal cancer [16]. Both the BM-MSCs of healthy donors engineered with IL-7 and IL-12 can enhance the antitumor cell reactivity of CAR-T cells. CAR-T cells also activate MSCs and release some cytokines that conversely activate CAR-T cells with extended persistence, amplification, killing and protection from AICD. Therefore, MSCs and CAR-T cells can mutually activate and improve each other’s function.

BM-MSCs from MM patients inhibit the lysis of native KHYG-1 NK cells in MM1.s and RPMI-8226 MM cell lines. However, the KHYG-1 NK cells engineered CD38-CAR increased the lysis of RPMI-8226 and MM1.s MM cells compared to MOCK control KHYG-1 NK cells. The lysis inhibition of BM-MSCs was significantly reduced in the presence of CD38-CAR- KHYG-1 NK cells in RPMI-8226 and MM1.s MM cells [129].

Altogether, these findings suggest that MSCs can regulate the proliferation and anti-cancer ability of CAR-related products. However, the different studies have obtained different outcomes. Thus, the different roles of MSCs in CAR-related products should be compared to select the most suitable MSCs.

### MSCs as CAR vehicle

MSCs can produce or overexpress a variety of proteins and exosomes continually or directly convey the cargo gene into the target cells for the treatment of clinical diseases, which indicates that MSCs can be used in gene delivery [130]. MSCs can deliver tumor necrosis factor-related apoptosis-inducing ligand (TRAIL). In vivo, MSCs could also protect against brainstem gliomas by delivering TRAIL [131]. A bifunctional MSC engineered with TRAIL and expressing the anti-GD2 CAR can enhance its antitumor abilities with site-specific targeting GD2 highly expressed in glioblastoma (GBM) [132]. Another study found that Ewing’s sarcoma cells can be recognized and killed by bifunctional MSCs engineered with TRAIL and truncated GD2-specific CAR in vitro. This anti-GD2 CAR also improved the persistence of MSCs and tumor targeting [133].

The MSCs cell line SCP-1 has been found to express bispecific antibodies (bsAbs) against CD33, and anti-CD3 secreted bsAb can effectively kill target cells by retargeted T cells, even at the lowest numbers of MSCs [134]. Further study showed that the bsAbs reinforced by costimulation with 4-1BBL strengthened the specific tumor cell killing of T cells. In addition, the activation markers CD25 and CD69 in CD4^+^ and CD8^+^ T cells can be upregulated via the two bsAb-producing MSCs lines. The use of the bsAb-producing MSC line with costimulation significantly increased the levels of TNF-α and IFN-γ secretion; conversely, stronger proliferation of bsAb-activated T cells was induced via these factors. However, the numbers of T cells did not increase in the presence of the bsAb-producing MSCs line without costimulation. These results show that the modified MSCs can continuously deliver bsAbs and constantly stimulate the expansion of T cells, which improves the specific killing of blasts.

Donor T cells attack host epithelial tissues in part via the interaction of T cell integrins with E-cadherin (Ecad) expressed on epithelia, which is one of the mechanisms for graft-versus-host disease (GVHD) after allogeneic hematopoietic stem cell transplantation (allo-HSCT). Ecad. CAR-MSCs with the CD28ζ signaling domain were generated to test the immunosuppressive function [135]. The antigen-stimulated Ecad. CAR-MSCs led to significant T cell suppression compared to unstimulated Ecad. CAR-MSCs and MSCs that were not transduced. In GVHD xenograft models, Ecad. CAR-MSCs significantly ameliorated weight loss, clinical GVHD score, T cell suppression and the induction of Treg production, which significantly improved the overall survival of mice. The levels of several serum cytokines, such as granulocyte colony stimulating factor, TNF-α and IL-10, were increased. The T cell inhibitory receptor of PD-1 and galectin-9 were upregulated.

In conclusion, these studies suggest that CAR-MSCs have strong anti-cancer and immunosuppression abilities mediated by gene delivery and the release of cytokines. However, the research on the anti-cancer and immunomodulatory roles of CAR-MSCs is limitted. Thus, more studies should be performed to explore the role of anti-cancer and immunomodulatory role of CAR-MSCs in different cancer cells and diseases. Moreover, the different structures of CAR-MSCs, especially the different costimulatory molecule involved in anti-cancer or immunomodulatory effects should be further explored.

### Conclusion and future developments

MSCs have a two-way effect on tissue damage repair, immune regulation, and tumor therapy outcome [136]. On one hand, MSCs promote the growth and metastasis of tumor cells. On the other hand, they can migrate to tumor tissues and inhibit their progression. Researchers have also found that MSCs can release soluble factors and exosomes, which may promote or inhibit cancers. Significantly, the CARs reformed by MSCs have stronger killing ability and can also improve the proliferation of MSCs and other immunoregulation cells, such as T cells (Table 5) [132–134]. Thus, MSCs can be used as important carriers for the delivery of anticancer biologics [136, 137].

**Table 5:** Role of CAR.MSCs

MSCs origin	CAR type	Cytokine secretion of T cells	T cell proliferation	MSCs proliferation	Tumor cell	Killing ability	Ref
Human adipose tissue	Anti-GD2 CAR lacks the intracellular signaling domain with mTRAIL	/	/	/	GBM cell lines (T98G, U87MG, and A172), primary C3c GBM cells	Robust cytotoxic effect	[132]
Human adipose tissue	Anti-GD2 CAR lacks the intracellular signaling domain with TRAIL	/	/	Prolonged persistence	ES cell lines(TC71, A673)	Significant antitumour activities	[133]
Human SCP-1 cell line	Anti-CD33-anti-CD3 bsAb with 4-1BBL	Increase the section of TNF-α and IFN-γ	Upregulated the activation markers CD69 and CD25, Pronounced proliferation	/	Human AML cell lines U937 and MOLM-13, OCI-AML3	Improvement of T-cell-mediated tumor cell killing	[134]

Notes: *MSCs* Mesenchymal stem cells, *CAR* Chimeric antigen receptor, *GBM* Glioblastoma, *ES* Ewing’s sarcoma, *AML* acute myeoloid leukemia, *TNF-α* Tumor necrosis factor-α, *IFN-γ* interferon-γ, *Ref.* Reference

MSCs can migrate to tumor sites and inflamed sites as damaged tissues expressing ligands or specific receptors that stimulate the trafficking, recruitment, adhesion and extravasation of MSCs [138]. MSCs-derived exosomes play vital roles in cancer therapy resistance, including resistance to immunotherapy, chemotherapy, radiotherapy, and targeted therapy [69, 139, 140]. MSCs-derived exosomes can be absorbed by different cell types and cause side effects by affecting nontargeted cells [141]. Thus, the modification of stromal cells themselves, rather than T cells, is an especially innovative approach to infiltrate tumors [142]. Therefore, the high tumor-homing capacity of MSCs has become an attractive vector for targeted cellular therapy. Numerous studies have shown that as a vector, MSCs have excellent function in killing tumor cells [142–144]. EVs derived from MSCs can also accumulate in cells, which might be a prerequisite for MSCs function [145–147]. The capability of the CARs originating from CAR-MSCs, which may be expressed in exosomes, is similar to that of CAR-related products [15, 133, 134].

The most common complication after allogeneic hematopoietic stem cell transplantation (allo-HSCT) is graft-versus-host disease (GVHD), which is also a cause of death or low quality of life [148–151]. MSCs have been extensively used in clinics, especially for GVHD prevention and treatment [152, 153]. The strong drugs used to treat GVHD affect the recovery of the immune system after allo-HSCT and may lead to disease relapse [154]. Therefore, the ideal method for the treatment of GVHD is to treat or prevent GVHD and simultaneously prevent disease relapse. Importantly, studies have shown that the preventative use of MSC-engineered CARs can not only effectively prevent the development of GVHD but also prevent the relapse of disease as a molecular policeman by MSCs themselves and the continuous release of exosomes, including those with CARs [15, 68–85, 155, 156]. These unique properties make MSCs an ideal vehicle for CARs in cellular immunotherapy. However, many changes remain for the clinical use of MSC-engineered CARs as a drug for cellular immunotherapy in the clinic.

First, the selection of the source of MSCs of origin is vitally important because MSCs can come from various tissues with different characteristics [157–161] (Table 6). MSCs should be easily obtained and isolated from normal tissues without increasing the pain among donors. The MSCs obtained from adipose-derived tissue and scaffolds in vaginal tissue cannot be used because of the tissue damage, pain and ethical issues in collecting these tissues. In this context, MSCs from the umbilical cord, umbilical cord blood, and placenta are the most suitable because of their easy accessibility and ease of turning trash into treasure. However, the characteristics of different tissue-derived MSCs with CARs need to be further compared to select the most ideal carrier. In addition, in vivo culture cannot cause the loss of MSC functionality [162].

**Table 6:** The biological characteristics of different tissue-derived mesenchymal stem cells

	Bone morrow	Adipose tissue	Perinatal tissues	hESC-derived
Accessibility	+	+	+ +	+ +
Pain	Yes	Yes	No	No
Ethical issues	Yes	Yes	No	No
morphology	Larger	Average	Smaller	Larger
Proliferation	+	+ +	+ + +	+ + +
Senescence	+ + +	+ +	+	+
Adipocytes differentiation potential	+	+ +	+	+
Osteoblasts differentiation potential	+ +	+	+	+
Chondroblasts differentiation potential	+ +	+	+ +	+
Secretion of Soluble Factors	+	+	+ +	+
Secretion of EVs and miRs	+	+	+	+
Immunomodulation	+ +	+	+ + +	+ + +

Notes: *miRs* Microribonucleic acids, *EVs* Extracellular vesicles. *hESC* Human embryonic stem cell

Second, the CAR type and structure of MSC-engineered CAR should be further explored in the context of different diseases. Normally, the major components of CARs are the intracellular signal transduction domain and extracellular tumor-antigen receptor. Several studies have used a truncated form of CAR, which lacks the signaling domains 4-1BB (CD137) and CD3-ζ, as the vector for MSCs. These truncated CAR-MSCs have no effect on tumor cell survival [132]. However, MSCs with both truncated CARs and mTRAIL can effectively kill tumor cells [132, 133]. Another study used anti-CD33-anti-CD3 as an MSCs CAR to strongly kill blast cells in acute myeloid leukemia. However, the ability to kill leukemia cells can significantly increase when CAR-MSCs are generated through costimulation with the 4-1BB ligand, not 4-1BB, which is usually used in CAR-T cells because 4-1BBL can crosslink with the 4-1BB molecule on activated T cells [134]. Therefore, researchers should generate different types of CARs according to the specific type of tumor.

Third, the effect of CAR on MSCs should be further investigated. Notably, the proliferation of CAR-MSCs both in vitro and in vivo is still not clear and should be further studied. Although one study showed that the persistence of MSCs generated with CARs is prolonged in an animal model [133], the persistence in patients is not clear at present because of the complexity of the body. The changes in the molecular genes or mutations of CAR-MSCs should be examined, which should not weaken the function of MSCs.

Fourth, the effect of CAR-MSCs- and CAR-MSCs-derived exosomes on other immune cells that may cooperate with each other to improve anticancer functions should be examined. Whether the exosomes released from CAR-MSCs can be detected and whether the role of CAR-MSCs-derived exosomes is similar to that of CAR-MSCs should be studied. In addition, whether exosomes, such as those with CARs can be absorbed and affect or strengthen the function of other immune cells, such as T cells, NK cells, DCs and macrophages, needs to be investigated [1, 113, 114].

Fifth, the numbers of CAR-MSCs that should be infused and the frequency of CAR-MSC treatment also need further investigation. The proposed number of MSC infusions used in clinics is 1 × 10^6^/kg once weekly [153]. The number of CAR-MSCs infusions used clinially is unknown because the time of CAR-MSCs persistence in patients or in vivo is unclear and should be further explored. Whether CAR-MSCs need to further express certain genes to promote the proliferation and persistence of CAR-MSCs is also important to decrease the number of infusions.

## Data Availability

Not applicable.
